# Magnetic Resonance Spectroscopy of Intra-axial Gliomas With Histopathological Correlation in a Tertiary Care Center of Eastern Nepal

**DOI:** 10.7759/cureus.54287

**Published:** 2024-02-16

**Authors:** Suraj Tiwari, Isha Gyawali

**Affiliations:** 1 Radiology, B.P. Koirala Institute of Health Sciences, Dharan, NPL; 2 Pathology, B.P. Koirala Institute of Health Sciences, Dharan, NPL

**Keywords:** brain metabolites, choline level and mr spectroscopy, lipid and lactate peak, gliomas, magnetic resonance spectroscopy (mrs)

## Abstract

Background and objective

Magnetic resonance spectroscopy (MRS) is a magnetic resonance imaging technique used to identify in vivo metabolites non-invasively within the tissue of interest. It plays an important role in diagnosing brain lesions, particularly tumors and infections. There are certain metabolites whose levels are increased or decreased in brain tumors, the ratios of which can also be used to grade the tumors as high- or low-grade. This study aimed to assess the spectrum of different metabolites in intraaxial gliomas using magnetic resonance spectroscopy and to assess the usefulness of their ratios for grading gliomas into high-grade and low-grade.

Methods

This descriptive cross-sectional study was performed in the radiology department of Nobel Medical College and Teaching Hospital, Biratnagar, Nepal over one year (September 2019 to September 2020). Thirty-five patients diagnosed as having intra-axial tumors were enrolled. After taking informed consent the examination findings were recorded in structured proforma. Siemens' 3 Tesla open magnet MAGNETOM Skyra (Siemens Healthineers AG, Munich, Germany) MR scanner was used to evaluate each patient. Data was analyzed using the software Statistical Package for Social Sciences (SPSS), version 26.0 (IBM Corp., Armonk, NY).

Results

Out of 35 patients scanned, 18 had high-grade glioma and 17 had low-grade glioma. High-grade glioma had a choline/creatine (Cho/Cr) ratio of 2.44 ± 0.78 and a choline/N-acetyl-aspartate (Cho/NAA) ratio of 2.05 ± 0.84. Low-grade glioma had a Cho/Cr ratio of 1.48 ± 0.50 and a Cho/NAA ratio of 1.41 ± 0.19. Fourteen out of eighteen high-grade gliomas had raised lipid/lactate peaks. The sensitivity, specificity, positive and negative predictive values (PPV and NPV), and accuracy for diagnosing high-grade glioma with a Cho/Cr ratio cut-off of 1.5 was 83.3 %, 82.4%, 83.3%,82.4 %, and 82.85% respectively.

Conclusion

MRS metabolite ratios can be used to diagnose and grade gliomas. Cho/Cr, Cho/NAA, and the presence or absence of lipid/lactate peak can significantly improve the sensitivity, specificity, predictive values, and accuracy of preoperative glioma grading when used in conjunction with conventional MRI.

## Introduction

Magnetic resonance spectroscopy (MRS) provides metabolites and biochemical information about tissues non-invasively in vivo [1]. The major metabolites commonly studied in brain lesions are N-acetyl aspartate (NAA), creatine (Cr), choline (Cho), myoinositol, glutamate and glutamine, alanine, lipid, and lactate. These metabolites reflect aspects of neuronal integrity, cell membrane proliferation or degradation, energy metabolism, and necrotic transformation of brain or tumor tissue. The metabolite level and ratios are used to diagnose and grade gliomas preoperatively [2]. This plays an important role in planning therapeutic approaches as low-grade gliomas may undergo either strict follow-up or surgery whereas high-grade gliomas are generally treated with surgery, followed by adjuvant radiation therapy and chemotherapy [3].

## Materials and methods

Thirty-five patients diagnosed as having intra-axial brain tumors on CT/MRI brain who later underwent surgery/biopsy were included. Ethical approval was obtained from the Institutional Review Committee of Nobel Medical College and Teaching Hospital, Biratnagar, Nepal (reference no. 298/2019). MRS was done on Siemens’ 3 Tesla open magnet MAGNETOM Skyra (Siemens Healthineers AG, Munich, Germany). Single-voxel MRS was performed with the positioning of the voxel on the enhancing edge of the tumor. Single voxel spectroscopy was applied for its excellent spectral quality and less spectral contamination. In the case of a non-enhancing tumor, the voxel was placed in the solid component of the lesion away from edema or necrosis. A single measurement of the voxel was taken. In case of unsatisfactory spectra because of the patient's motion, the measurement was repeated. Pulse gating was used to lessen pulsation artifacts. The pulse sequence used was STEAM (stimulated echo acquisition mode) with an echo time (TE) of 35 ms and repetition time (TR) of 1700 ms. Localized shimming of the magnetic field was done followed by phase correction and water signal suppression. The voxel size used was 1.5 × 1.5 × 1.5 cm^3^ with a field of view (FOV) of 160 × 160 × 15 mm^3^. The integral values of the different metabolites were documented and their ratios were calculated. The histopathological report was collected and then correlated with MRS findings.

Inclusion and exclusion criteria

Patients, newly diagnosed or referred cases, showing features of intra axial gliomas on CT/MRI were included in this study. Patients from all age groups and sexes were included. Patients with extra-axial brain tumors were excluded. The patient who previously underwent surgery, chemotherapy, or radiotherapy were also excluded.

Statistical analysis

The collected data were entered in Statistical Package for Social Sciences (SPSS), version 26.0 (IBM Corp., Armonk, NY) for statistical analysis. Pearson Chi-square test and Fisher exact test were applied to find out significant differences between dependent and independent variables at 95% CI (Confidence Interval) where the p-value was less than 0.05. Predictive values, sensitivity, specificity, and accuracy of MRS diagnosis were calculated considering histopathology as the gold standard diagnostic test.

Study definition

MR spectroscopy was used as a diagnostic test for diagnosing brain tumors. An increase in choline peak at 3.2 ppm, myoinositol peak at 3.6 ppm, lipid/lactate (lip/lac) peak at 0.9-1.4 ppm, and reduced NAA peak at 2.0 ppm was considered significant for diagnosing brain tumors. We reported brain tumors as a high grade if there was an increase in the Cho/Cr ratio of more than 1.5 and the Cho/NAA ratio of more than 1.6 and low grade if the Cho/Cr ratio was less than 1.5 and the Cho/NAA ratio of less than 1.6. This value was used as a threshold value to increase the sensitivity of detecting brain tumors. Elevation of lip/lac peak was considered significant for high-grade glioma.

## Results

Out of 35 patients studied 20 (57.14%) were male, while 15 (42.86%) were female (Table 1). The patient's ages ranged from 1-80 years with the majority of the patients between 31 to 40 years of age (Table 2). Eighteen (51.43%) patients had high-grade glioma and 17 (48.57%) patients had low-grade glioma. All the cases showed elevated choline and reduced NAA. The lip/lac peak was elevated in 51.42% of cases. High-grade glioma had a Cho/Cr ratio of 2.44 ± 0.78 and a Cho/NAA ratio of 2.05 ± 0.84. Low-grade glioma had a Cho/Cr ratio of 1.48 ± 0.50 and a Cho/NAA ratio of 1.41 ± 0.19.

**Table 1:** Distribution of sample according to sex.

Gender	No. of cases	Percentage
Male	20	57.14
Female	15	42.86
Total	35	100.0

**Table 2:** Distribution of sample according to age.

Age (in years)	No. of cases	Percentage
1-10	3	8.57
11-20	3	8.57
21-30	3	8.57
31-40	9	25.71
41-50	6	17.14
51-60	7	20
61-70	3	8.57
71-80	1	2.86
Total	35	100

There is a significant association between elevated lip/lac peak with high-grade glioma as shown in Table 3. The sensitivity, specificity, positive predictive value (PPV), negative predictive value (NPV), and diagnostic accuracy of elevated lip/lac peak in diagnosing high-grade glioma were 83.33%, 82.35%, 83.33%, 82.35%, and 82.86% respectively.

**Table 3:** Confusion matrix of elevated lip/lac with histopathological diagnosis. Lip/lac: Lipid lactate, HGG: High-grade glioma, LGG: Low-grade glioma. The data has been represented as N with p <0.05 considered as significant.

		Histopathology			
		HGG	LGG	Total	Fisher exact test
Lip/Lac	HGG	15	3	18	p=0.000
	LGG	3	14	17	
	Total	18	17	35	

There is a significant association between elevated Cho/Cr and histopathological findings for high-grade glioma as shown in Table 4. The sensitivity, specificity, PPV, NPV, and diagnostic accuracy of the Cho/Cr ratio in diagnosing high-grade glioma was 83.33%, 82.35%, 83.33%, 82.35%, and 82.86% respectively. 

**Table 4:** Confusion matrix of Cho/Cr ratio with histopathological diagnosis with ratio > 1.5 considered positive for HGG. Cho/Cr: Choline/creatine, HGG: High-grade glioma, LGG: Low-grade glioma. The data has been represented as N with p <0.05 considered as significant.

		Histopathology			
		HGG	LGG	Total	Fisher exact test
Cho/Cr	HGG	15	3	18	p = 0.000
	LGG	3	14	17	
	Total	18	17	35	

There is a significant association between the increased Cho/NAA ratio and high-grade glioma as shown in Table 5. The sensitivity, specificity, PPV, NPV, and diagnostic accuracy of the Cho/NAA ratio in diagnosing high-grade glioma was 77.78%, 82.35%, 82.35%, 77.78%, and 80% respectively.

**Table 5:** Confusion matrix of Cho/NAA ratio with histopathological diagnosis with ratio > 1.60 considered positive for HGG. Cho/NAA: Choline/n-acetyl aspartate, HGG: High-grade glioma, LGG: Low-grade glioma. The data has been represented as N with p <0.05 considered as significant.

		Histopathology			
		HGG	LGG	Total	Fisher exact test
Cho/NAA	HGG	14	3	17	p = 0.001
	LGG	4	14	28	
	Total	18	17	35	

**Figure 1:**
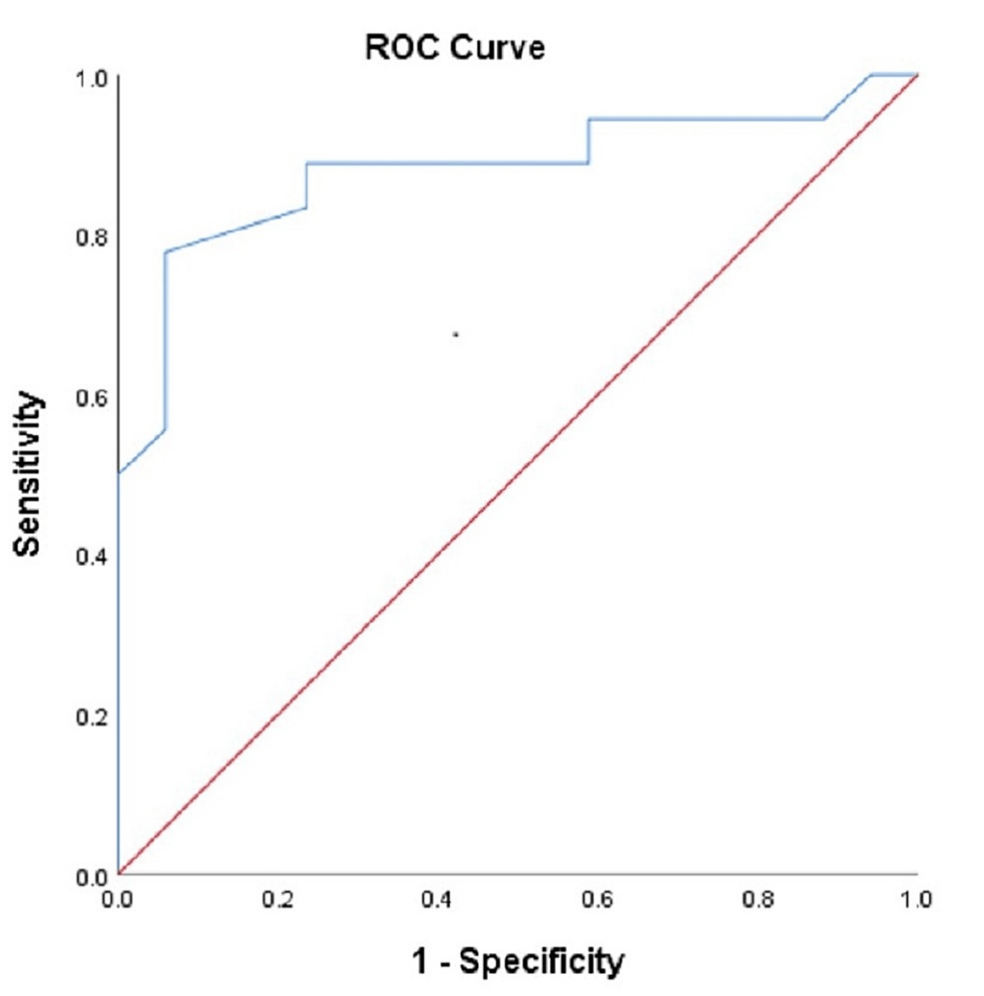
ROC curves for glioma grading with Cho/Cr ratio. Cho/Cr ratio: Choline/creatine, ROC: Receiver operating characteristic. The red line represents the main diagonal line and the blue line represents the ROC curve. The area below the blue line represents the area under the curve.

**Figure 2:**
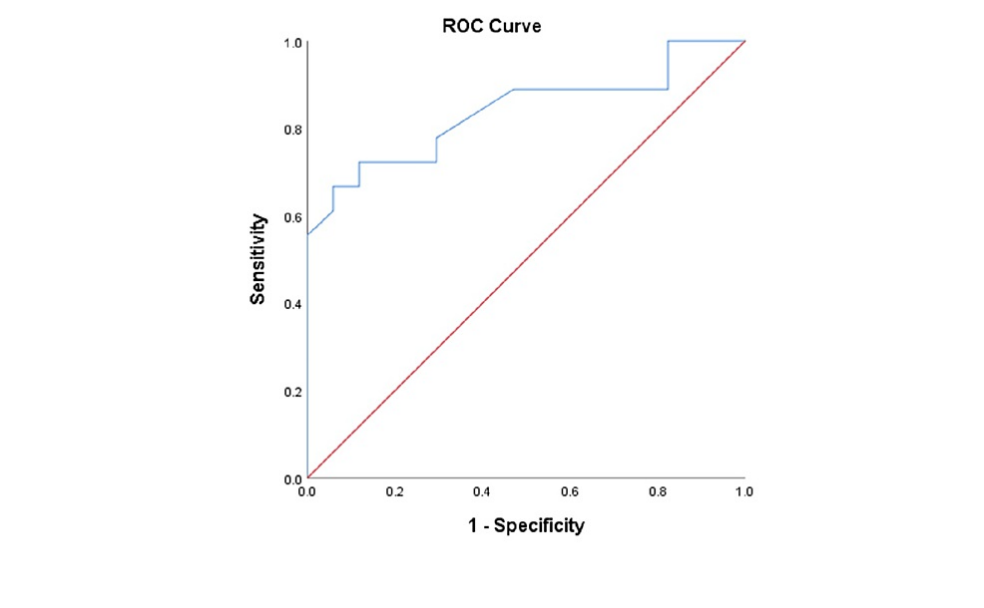
ROC curves for glioma grading with Cho/NAA ratio. Cho/NAA: Choline/N-acetyl aspartate, ROC: Receiver operating characteristic. The red line represents the main diagonal line and the blue line represents the ROC curve. The area below the blue line represents the area under the curve.

Figure 1 shows the receiver operating characteristic curves and area under the curve (AUC) for glioma grading with Cho/Cr. Figure 2 shows the receiver operating characteristic curves and AUC for glioma grading with Cho/NAA. The area under the curve for glioma grading with Cho/Cr and Cho/NAA was 0.881 and 0.838 respectively as shown in Figure 1 and Figure 2.

## Discussion

**Figure 3:**
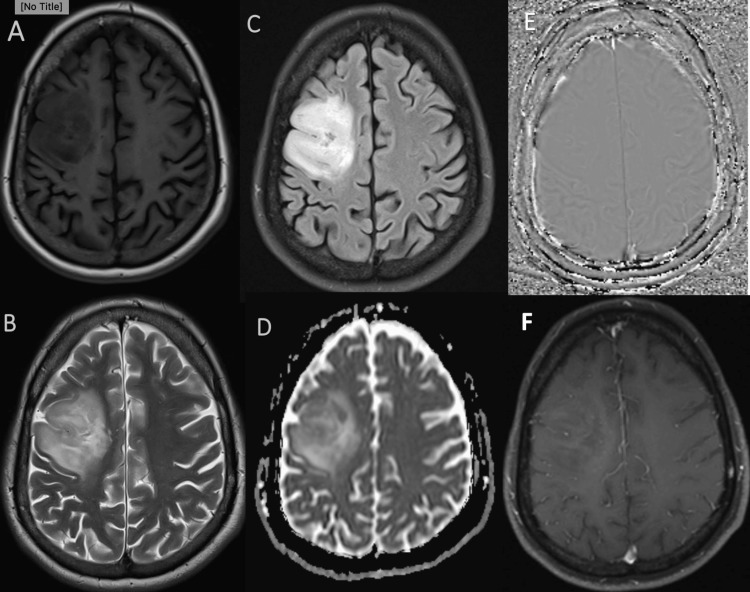
MRI of right frontal lobe diffuse astrocytoma showing hypointense signal on T1WI (A), hyperintensity seen on T2WI/FLAIR image (B, C). No diffusion restriction on the ADC image (D), no calcification/blood products in the filtered phase image (E), and no enhancement in the post-contrast image (F). ADC: Apparent diffusion coefficient, FLAIR: Fluid attenuated inversion recovery, T1WI: T1 weighted image, T2WI: T2 weighted image.

**Figure 4:**
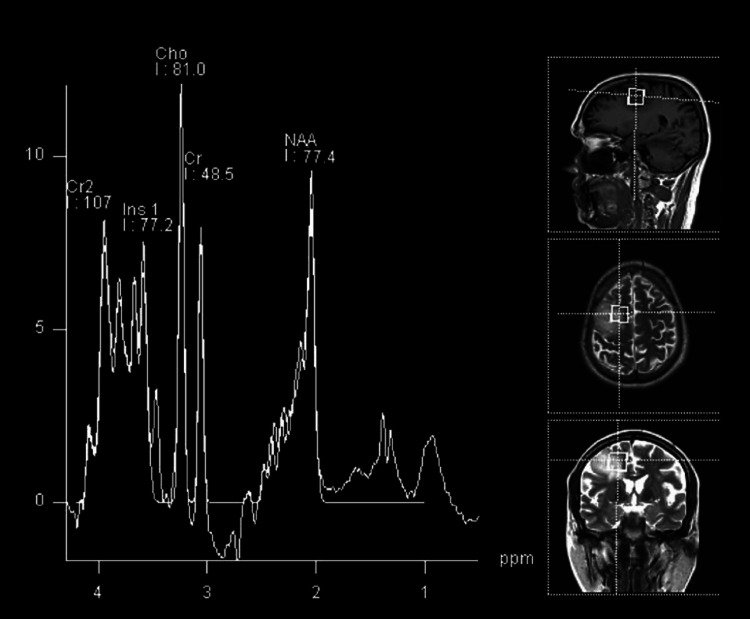
MRS of low-grade glioma showing mildly elevated choline and reduced NAA. MRS spectrum with the x-axis representing metabolites at ppm and the y-axis representing intensity. Choline peak at 3.2 ppm, NAA peak at 2.0 ppm, and creatine peak at 3.0 ppm. MRS: Magnetic resonance spectroscopy, NAA: N-acetyl aspartate

This study was performed to determine the level of metabolites for diagnosing and grading gliomas which was confirmed by histopathology. Low-grade gliomas are generally characterized by a relatively high concentration of N-acetyl aspartate, low levels of choline, and absence of lactate and lipids [4]. MR image and MRS of low-grade glioma are shown in Figure 3 and Figure 4.

**Figure 5:**
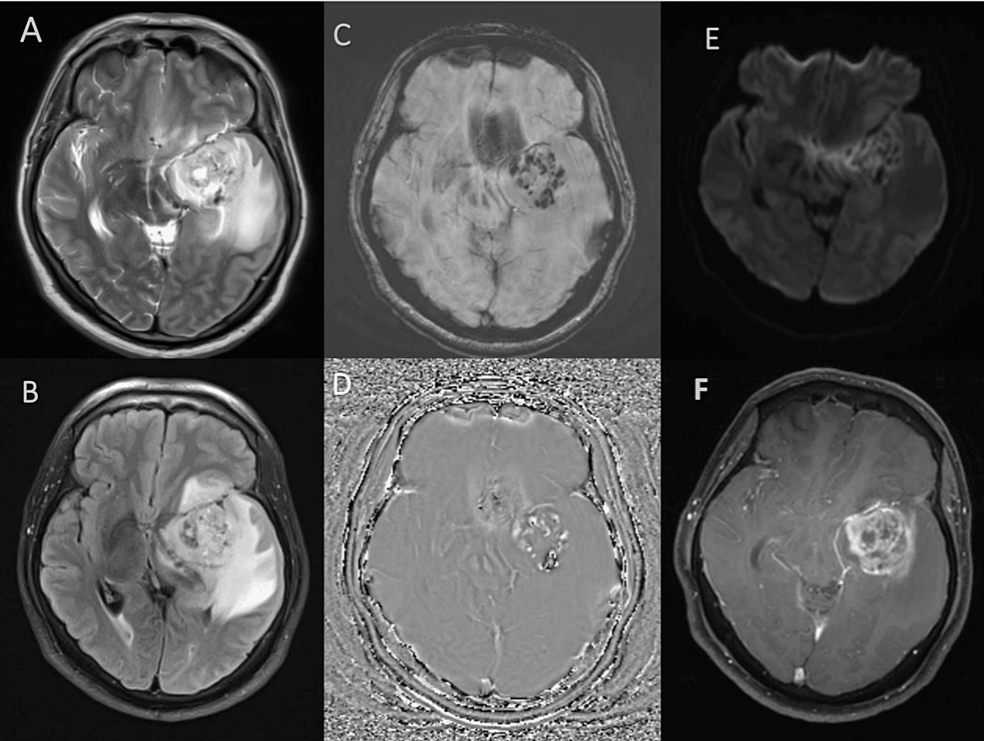
MRI image of left temporal glioblastoma with significant mass effect, areas of diffusion restriction, punctate areas of hemorrhage, moderate perilesional edema, and thick peripheral enhancement. (A: T2WI, B: FLAIR, C: SWI magnitude image, D: Filtered phase image, E: DWI, F: T1FS post-contrast image.) DWI: Diffusion-weighted imaging, FLAIR: Fluid attenuated inversion recovery, SWI: Susceptibility weighted imaging, T1FS: T1 weighted fat-saturated image, T2WI: T2 weighted image

**Figure 6:**
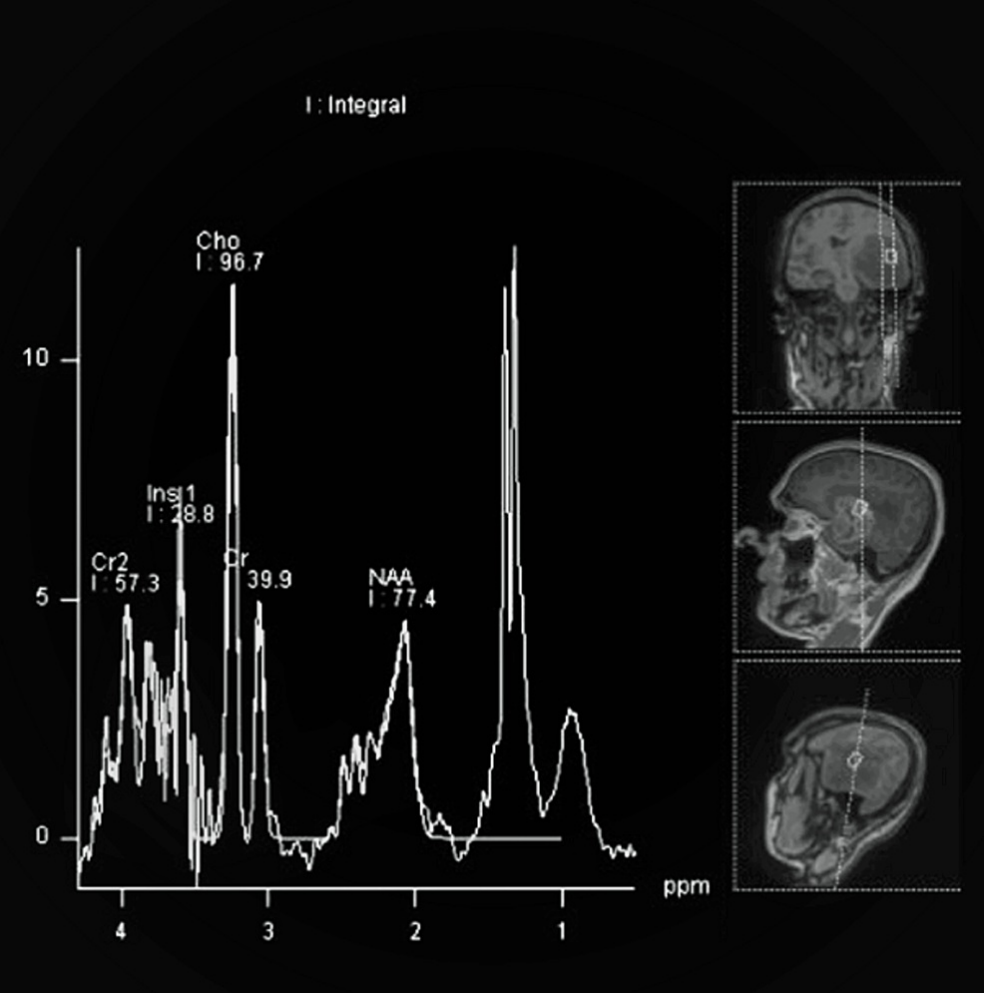
MRS of left temporal high-grade glioma showing markedly elevated choline, reduced NAA, and markedly elevated lip/lac peak. MRS spectrum with the x-axis representing metabolites at ppm and the y-axis representing intensity. Choline peak at 3.2 ppm, NAA peak at 2.0 ppm, creatine peak at 3.0 ppm, and elevated lip/lac peak at 0.9-1.4 ppm. MRS: Magnetic resonance spectroscopy, Lip/lac: Lipid/lactate, NAA: N-acetyl aspartate.

High-grade glioma shows elevated lipid lactate peak in addition to elevated choline and reduced NAA peak as shown in Figure 5 and Figure 6.

Our study showed an elevation of choline, reduction of creatine, and reduction of NAA in all the cases of brain tumors which was consistent with Hourani et al. and Alshammari et al. [5,6]. Cho/Cr ratio and Cho/NAA ratio were increased in all of our cases and the ratio increased with the increasing grade of glioma. This finding was consistent with the study by Magalhaes et al., Shakir et al., and Kousi et al. [7-9]. 

In our study, the sensitivity and specificity of glioma grading with Cho/Cr and Cho/NAA was within the range as reported by a meta-analysis of 30 studies done by Wang et al. [10]. Elevated lipid and lactate peak was seen in 15 out of 18 high-grade gliomas and were statistically significant in diagnosing high-grade glioma, consistent with Fawzy et al. and Nakamura et al. [11,12].

Our study showed a statistically significant result of glioma grading with Cho/Cr and Cho/NAA ratio which was consistent with Zeng et al. [13]. The mean Cho/Cr ratio in high-grade and low-grade glioma in our study was comparable to that of Kousi et al. at short TE [9]. The diagnostic accuracy of Cho/Cr and Cho/NAA ratios in glioma grading was 82.86 and 80% which was in agreement with Hamsini et al. [14]. The receiver operating characteristic curve analysis of the Cho/Cr ratio yielded an AUC of 0.881 whereas that of Cho/NAA yielded an AUC of 0.838. Despite no significant difference in diagnostic accuracy between the metabolite ratios, diagnostic accuracy using the Cho/Cr ratio was slightly better than that of Cho/NAA in concordance with Liu et al. [15]. Wang et al. confirmed overall accuracy in differentiating high- and low-grade gliomas was greatest with the Cho: NAA, with an area under the curve of 0.87 [10]. However, in our study, the accuracy of glioma grading was greatest with both the Cho/Cr ratio and elevation of the lip/lac peak.

Limitations

This study classified brain tumors according to the World Health Organization (WHO) 2016 classification of tumors of the central nervous system. So, the incorporation of molecular markers for diagnosis compliant with WHO CNS 5 and division of gliomas into adult type and pediatric type is lacking. This study was performed in a short duration of time with a limited number of patients. A longer duration of study with a larger number of cases would provide more accurate results. Since single-voxel spectroscopy was used in this study the area with a higher concentration of metabolites could have been missed in comparison to multi-voxel spectroscopy where multiple spectra are demonstrated. This could have affected the metabolite ratio. 

## Conclusions

Magnetic resonance spectroscopy (MRS) is a valuable tool for diagnosing and grading intra-axial gliomas. The levels and ratios of metabolite peaks provide useful information for differentiating between low-grade and high-grade gliomas. The ratios of Cho/Cr and Cho/NAA were found to be valuable in diagnosing and grading gliomas, with an increasing ratio correlating with higher grades of glioma. The sensitivity, specificity, and diagnostic accuracy of using these ratios were consistent with previous studies, highlighting the reliability of MRS in glioma grading. Furthermore, the presence of an elevated lipid and lactate peak was observed in high-grade gliomas, providing additional evidence for diagnosing high-grade gliomas. By providing valuable insights into tumor grade, MRS can aid clinicians in determining the appropriate treatment strategy for patients with intra-axial gliomas.
